# “3‐Day Surprise Question” to predict prognosis of advanced cancer patients with impending death: Multicenter prospective observational study

**DOI:** 10.1002/cam4.3689

**Published:** 2020-12-21

**Authors:** Tomoo Ikari, Yusuke Hiratsuka, Takuhiro Yamaguchi, Isseki Maeda, Masanori Mori, Yu Uneno, Tomohiko Taniyama, Yosuke Matsuda, Kiyofumi Oya, Keita Tagami, Akira Inoue

**Affiliations:** ^1^ Department of Palliative Medicine Tohoku University School of Medicine Sendai Japan; ^2^ Division of Biostatistics Tohoku University School of Medicine Sendai Japan; ^3^ Department of Palliative Care Senri‐Chuo Hospital Toyonaka Japan; ^4^ Division of Palliative and Supportive Care Seirei Mikatahara General Hospital Hamamatsu Japan; ^5^ Department of Therapeutic Oncology Graduate School of Medicine Kyoto University Kyoto Japan; ^6^ Department of Oncology and Palliative Medicine Mitsubishi Kyoto Hospital Kyoto Japan; ^7^ Palliative Care Department St. Luke's International Hospital Tokyo Japan; ^8^ Aso Iizuka Hospital, Transitional and Palliative Care Iizuka ciyu Japan

**Keywords:** advanced cancer, end‐of‐life care, impending death, predict prognosis, surprise question

## Abstract

**Background:**

The study aimed to clarify the efficacy of the “3‐Day Surprise Question (3DSQ)” in predicting the prognosis for advanced cancer patients with impending death.

**Patients and Methods:**

This study was a part of multicenter prospective observational study which investigated the dying process in advanced cancer patients in Japan. For patients with a Palliative Performance Scale ≤20, the 3DSQ “Would I be surprised if this patient died in the next 3 days?” was answered by their physicians. In addition to the sensitivity and specificity of the 3DSQ, the characteristics of patients who survived longer than expected were examined via multivariate analysis.

**Results:**

Among the 1896 patients enrolled, 1411 were evaluated. Among 1179 (83.6%) patients who were classified into the “Not surprised” group, 636 patients died within 3 days. Among 232 (16.4%) patients of “Yes surprised” group, 194 patients lived longer than 3 days. The sensitivity, specificity, positive predictive value, and negative predictive value of the 3DSQ were 94.3% (95% confidence interval [CI]: 92.7% to 95.8%), 26.3% (95% CI: 24.8% to 27.6%), 53.9% (95% CI: 53.0% to 54.7%), and 83.6% (95% CI: 78.7% to 87.7%), respectively. Multivariate analysis showed palpable radial artery, absent respiration with mandibular movement, SpO_2_ ≥ 90%, opioid administration, and no continuous deep sedation as characteristics of patients who lived longer than expected.

**Conclusions:**

The 3‐Day Surprise Question can be a useful screening tool to identify advanced cancer patients with impending death.

## Graphical abstract

Survival prediction in cancer patients with impending death is important for patients, families, and physicians. The 3‐Day Surprise Question can be a useful screening tool to identify advanced cancer patients with impending death.

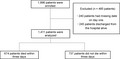

## 1 INTRODUCTION

Survival prediction in cancer patients with impending death is important for patients, families, and physicians. This sets the timeline to achieving a “good death”, where the patient's final wishes are accommodated and harmful interventions are stopped.^1^ The Palliative Prognosis Score (PaP score), Palliative Prognostic Index (PPI), Prognosis in Palliative care Study predictor models (PiPS models), and Surprise Question were helpful for prognosis prediction.^2^, ^3^, ^4^, ^5^, ^6^ Particularly, the Surprise Question (SQ), “Would I be surprised if this patient died in the next 12 months?,” was simple, sensitive, and specific. Thus, it was useful for predicting the prognosis of cancer patients in the next 12 months. Although these tools can predict the prognosis in a weekly, monthly, or yearly basis, a new prognostic tool is necessary to predict the prognosis patients impending death within days.

Prediction of a cancer patient's prognosis within 3 days may be helpful in accommodating the patient's final wishes while still receiving high quality end‐of‐life care.^7^ Previous studies attempted to predict a cancer patient's prognosis within 3 days. Hui et al., used physical signs and showed that Cheyne–Stokes breathing, pulselessness of the radial artery, peripheral cyanosis, drooping of nasolabial folds, and Palliative Performance Scale (PPS) ≤20 were predicted the prognosis within 3 days. These physical signs had high specificity but low sensitivity for death within 3 days.^8^, ^9^ It is necessary to develop highly‐sensitive prognostic tools. In a previous study, Hamano et al., reported that a 7‐day surprise question, “Would I be surprised if this patient died in the next 7 days?,” and a 30‐day surprise question, “Would I be surprised if this patient died in the next 30 days?,” were highly sensitive tools for predicting prognosis at 7 and 30 days, respectively.^10^ With this, we considered the 3‐Day Surprise Question (3DSQ) “Would I be surprised if this patient died in the next 3 days?” could be as a high sensitive prognostic tool as well as them.

The purpose of this study was to clarify the usefulness of the 3DSQ in predicting the prognosis for advanced cancer patients with impending death. In addition, we aimed to estimate the characteristics that led physicians to incorrectly predict that patients live longer.

## 2 METHODS

### 2.1 Participants

This study was a secondary analysis from a multicenter prospective observational study, which was conducted to uncover the dying process and end‐of‐life care in advanced cancer patients in Japan. The study was named East‐Asian collaborative cross‐cultural Study to Elucidate the Dying process (EASED). Consecutive eligible patients were enrolled if they had been newly referred to the participating Palliative Care Units (PCUs) during the study period. Observations were implemented within daily medical practice.

Adult patients (aged 18 years or older) who were diagnosed with locally extensive or metastatic cancer and admitted to PCUs were included in this study. On the contrary, patients who were scheduled for discharge within a week and refused approval were excluded from this study.

All data were prospectively recorded by physicians on a structured data‐collecting sheet made for this study which was piloted prior to study initiation.

### 2.2 Data collection

We analyzed the required data for our analysis from EASED.

We collected information on the patients’ characteristics at hospitalization (age, sex, primary cancer site, metastasis, complication, and medical treatment histories, Eastern Cooperative Oncology Group performance status [ECOG PS] and PPS) and treatment received during hospitalization (oxygen therapy, presence of opioid administration, sedation). In addition, we also collected the physical signs (Richmond Agitation Sedation Scale score [RASS], response to verbal stimuli, response to visual stimuli, peripheral cyanosis, pulse of radial artery, respiration with mandibular movement, bronchial secretions, and dysphagia of liquids), vital signs (body temperature, oxygen saturation of peripheral artery, and respiratory rate), and patients’ clinical symptoms (pain, dyspnea, fatigue, edema, pleural effusion, and ascites) on the first day when each patient had PPS ≤20. Patients’ clinical symptoms (pain, dyspnea, and fatigue) were evaluated by Integrated Palliative care Outcome Scale [IPOS]. The above factors were selected as representative prognostic factors, which the authors considered to be prognostic in daily clinical practice.

Physicians were asked the 3DSQ, “Would I be surprised if this patient were to die within 3 days?,” on the first day, when each patient had PPS ≤20. The physicians answered the 3DSQ with “Not surprised” or “Surprised”.

### 2.3 Data analysis and statistics

The patients were followed up until death. We set “day one” as the first day when each patient had PPS ≤20, and we defined "death within 3 days" as death from day 1 to day 3.

The patients were initially classified into “Not surprised” and “Surprised” groups based on the 3DSQ response of their physicians and the patient's status (alive or dead) on day 3. Moreover, sensitivity and specificity as well as positive and negative predictive values were calculated using simple statistical analysis by 2 × 2 contingency tables.

Second, to identify the factors of all patients who lived longer than 3 days contrary to the expectations of their physicians, we divided the patients into four groups. Group A consisted of patients whose physicians answered “not surprised” and actually died within 3 days. Group B (defined as “lived longer group”) were patients whose physicians answered “not surprised” and did not actually die within 3 days, Group C consisted of patients whose physicians answered “surprised” and actually died within 3 days. Lastly, Group D consisted of patients whose physicians answered “surprised” and did not actually die within 3 days. We then divided the four groups into two groups in order to analyze the data. The “lived longer group” consisted of patients whose physicians answered "not surprised" and did not actually die within 3 days. The “other group” consisted of patients whose physicians’ expectations were correct or who died earlier than expected. In other words, the "other group" is the sum of groups A, C, and D.

Third, we performed a Cochran–Armitage trend test for ordinal variables and a Fisher's exact test for categorical variables to identify the factors related to the "Lived longer group".

Fourth, to identify the factors associated with "Lived longer group," we performed a multivariate logistic regression analysis, using seven typical signs that were previously used to predict prognosis (RASS, Dyspnea, Pain, Dysphagia of liquids, Edema, Delirium, Prediction of prognosis at PPS ≤20) and signs that were significant in univariate analysis. A *p* value less than 0.05 was considered statistically significant in this study.

Statistical analysis was performed with JMP pro version 14 for Windows (SAS). In addition, all statistical analyses were performed with the advice of the Statistics Department.

## 3 RESULTS

### 3.1 Patients’ characteristics

**FIGURE 1:**
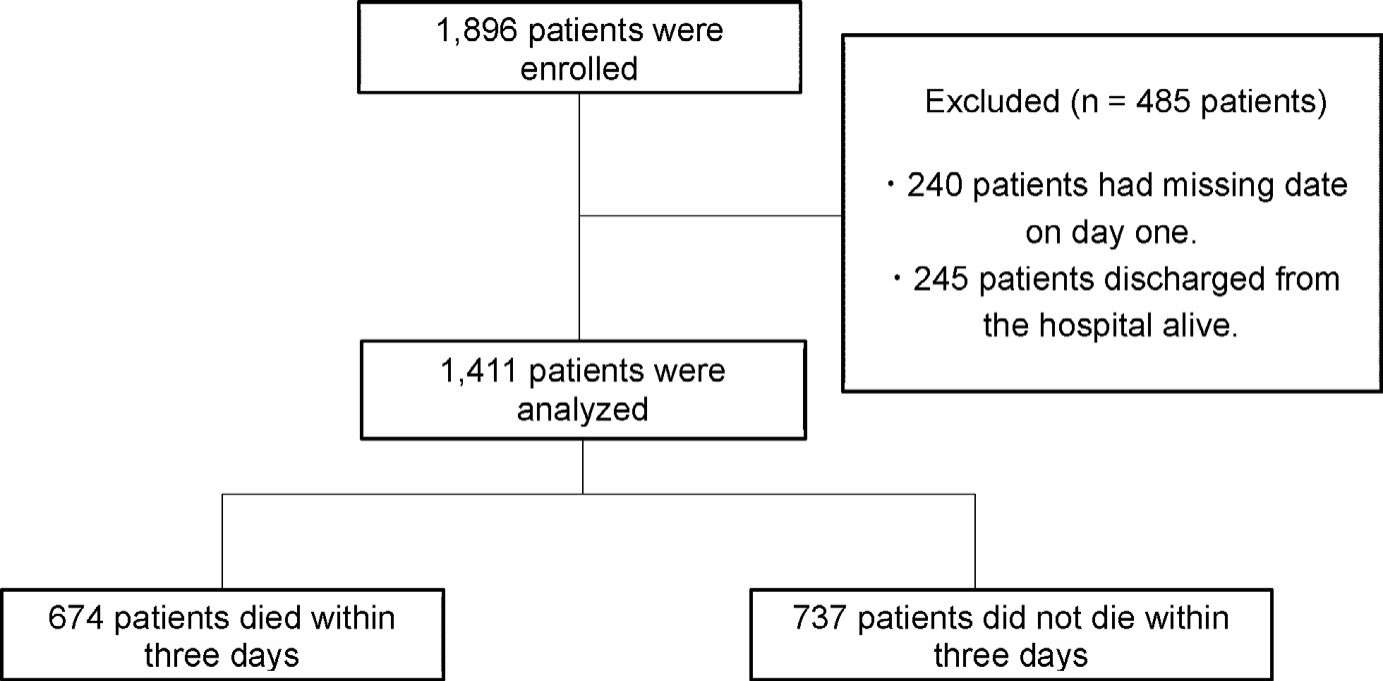
Patients selection for this study

A total of 1,896 patients were enrolled from 22 PCUs in Japan from January 2017 to December 2017. The median length of hospitalization period was 16 days (range 0–375 days). The overall median survival was 17 days (range 0–375 days). A total of 485 patients were excluded because 240 patients did not have an exact date for day one and 245 patients were discharged from the hospital alive. Thus, a total of 1,411 patients were evaluated (Figure 1).

Table 1 shows the baseline characteristics of the analyzed 1411 patients. The mean [SD] age was 72.6 [12.2] (range 25–100), and 716 (50.7%) patients were male. The primary sites were more commonly found in the lungs (17.1%), upper gastrointestinal tract (14.6%), lower gastrointestinal tract (13.1%), and pancreas (10.3%). The average prognosis for patients predicted by physicians on day one was 7.2 days.

**TABLE 1:** Patient's characteristics of the analyzed 1411 patients at hospitalize

Total (*n* = 1411)			
Characteristics	No. (%)	Characteristics	No. (%)
Age(years), mean (SD) [range]	72.6 (12.2) [25–100]	Comorbidity	
Male sex	716 (50.7)	Cardiovascular	82 (5.8)
Primary cancer site		Cerebrovascular	112 (7.9)
Lung	242 (17.1)	Lung	83 (5.8)
Stomach/Esophagus	207 (14.67)	Diabetes mellitus	184 (13.0)
Colon/Rectum	186 (13.1)	Dementia	125 (8.8)
Prostate/Bladder/Kidney/Testis	102 (7.2)	Eastern cooperative oncology group performance status	
Pancreas	146 (10.3)		
Ovary/Uterus	82 (5.8)	0–1	8 (0.6)
Liver/Biliary system	121 (8.5)	2	79 (5.6)
Others	325 (23.0)	3	549 (38.9)
Metastatic site		4	775 (54.9)
Liver	565 (40.0)	Palliative Performance Scale	
Bone	380 (26.9)	20 or less	326 (23,1)
Lung	530 (37.5)	30	296 (21.0)
Cancer treatment		40	410 (29.0)
Surgery	594 (42.0)	50	281 (20.0)
Chemotherapy	866 (61.3)	60 and above	98 (6.9)
Hormonal therapy	14 (0.9)		
Radiation therapy	11 (0.7)	Prediction of prognosis (days), mean(SD) [range]	27.1 (22.9) [0–180]

Abbreviation: SD, standard deviation.

Table 2 shows a 2 × 2contingency table. For 1179 (83%) of the patients, physicians answered that they would not be surprised if the patient died within 3 days. The sensitivity of the 3DSQ ‘‘not surprised” result showed a sensitivity of 94.3% (95% confidence interval [CI]: 92.7% to 95.8%) and specificity of 26.3% (95% CI: 24.8% to 27.6%). The positive predictive value was 53.9% (95% CI: 53.0% to 54.7%), the negative predictive value was 83.6% (95% CI: 78.7% to 87.7%) and the accuracy was 58.8% (95% CI: 57.2% to 60.2%).

**TABLE 2:** 2 × 2 contingency table

Group	Death within 3 days	Not death within 3 days	
Not surprised	Group A; 636	Group B; 543	

Surprised	Group C; 38	Group D; 194	

Sensitivity	94.3% (95% CI: 92.7%–95.8%)	Positive predictive value	53.9% (95% CI: 53.0%–54.7%)
Specificity	26.3% (95% CI: 24.8%–27.6%)	Negative predictive value	83.6% (95% CI: 78.7%–87.7%)

Group A: "Patients who physicians answered "not surprised" and actually die within three days". Group B: "Group that lived longer than expected (defined as "Lived longer group"): patients who physicians answered "not surprised" and did not actually die within three days". Group C: "Patients who physicians answered "surprised" and actually die within three days". Group D: "Patients who physicians answered "surprised" and did not actually die within three days".

Abbreviation: CI, Confidence interval.

The results of all variables for which univariate analysis was performed are shown in Table S1. Table 3 summarized 11 variables which associated with the factors related to the "Lived longer group" in Table S1. These variables included a decreased response to verbal stimuli (*p* = 0.001), decreased response to visual stimuli (*p* = 0.001), peripheral cyanosis (*p* = 0.004), radial artery (*p* < 0.001), respiration with mandibular movement (*p* < 0.001), increased bronchial secretions (*p* = 0.009), respiratory rate (*p* = 0.041), SpO_2_ (*p* < 0.001), opioid administration (*p* = 0.002), continuous deep sedation (*p* = 0.005), and infusion therapy (*p* = 0.035).

**TABLE 3:** The results of univariate analysis which associated with the factors related to the "Lived longer group"

Total (*n* = 1411)		Total	Lived longer group		Other group		*p*
Variables		*n*	*n*	%	*n*	%	
Decreased response to verbal stimuli	No	1172	477	40.7	695	59.3	0.002*
	Yes	224	66	29.5	158	70.5	
Decreased response to visual stimuli	No	1012	420	41.5	592	58.5	0.001*
	Yes	384	123	32	261	68	
Peripheral cyanosis	No	1146	466	40.7	680	59.3	0.004*
	Yes	250	77	30.8	173	69.2	
Pulselessness of radial artery	No	1327	531	40	796	60	<0.001*
	Yes	69	12	17.4	57	82.6	
Respiration with mandibular movement	No	1346	540	40.1	806	59.89	<0.001*
	Yes	50	3	6	47	94	
Increased bronchial secretions	No	1067	435	40.8	632	59.2	0.009*
	Yes	329	108	32.8	221	67.2	
Respiratory rate	24 times or less per minute	1100	449	40.8	651	59.2	0.041*
	25 times or more per minute	76	22	29	54	71	
SpO2	90% and above	123	27	22	96	78	<0.001*
	89% or less	1162	492	42.2	674	57.8	
Presence of opioid administration	No	324	150	46.3	174	53.7	0.002*
	Yes	1072	393	36.7	679	63.3	
Continuous deep sedation	No	1349	534	39.6	815	60.4	0.006*
	Yes	47	9	19.2	38	80.8	
Infusion therapy	No	514	181	35.2	333	64.8	0.035*
	Yes	882	362	42	520	58	

* A *p* value less than 0.05 was considered statistically significant.

Table 4 listed the results of the multivariate analysis. Five factors were reported to be independently associated with the "Lived longer group". These factors were the radial artery (palpable vs. pulselessness) (odds ratio [OR] 2.29; 95% CI: 1.13 to 4.64; *p* = 0.021), respiration with mandibular movement (absent vs. present) (OR 6.76; 95% CI: 2.02 to 22.50; *p* = 0.002), SpO_2_ (≥90% vs. <89%) (OR 1.93; 95% CI: 1.19 to 3.10; *p* = 0.007), opioid administration (present vs. absent) (OR 1.48; 95% CI: 1.08 to 2.01; *p* = 0.013), and continuous deep sedation (no vs. yes) (OR 2.64; 95% CI: 1.12 to 6.17; *p* = 0.017).

**TABLE 4:** The results of multivariate analysis which associated with the factors related to the "Lived longer group"

Variables		OR(95%CI)	*p*
Radial artery	Palpable	2.29 (1.13–4.64)	0.021*
	Pulselessness	ref	
Respiration with mandibular movement	Absent	6.76 (2.02–22.5)	0.002*
	Present	ref	
SpO_2_	90% and above	1.93 (1.19–3.10)	0.007*
	89% or less	ref	
Opioid administration	Present	1.48 (1.08–2.01)	0.013*
	Absent	ref	
Continuous deep sedation	No	2.64 (1.12–6.17)	0.017*
	Yes	ref	

Abbreviations: CI, Confidence interval; OR, Odds ratio.

* A *p* value less than 0.05 was considered statistically significant.

## 4 DISCUSSION

In this study, we showed that the 3‐Day Surprise Question was a viable screening tool in identifying advanced cancer patients impending death, especially within 3 days. Moreover, we showed five variables associated with the patients who were more likely to survive for more than 3 days, contrary to the physician's prediction. These variables were a palpable radial artery, absent respiration with mandibular movement, SpO_2_ ≥ 90%, opioid administration, and no continuous deep sedation.

The sensitivity and specificity of the 3DSQ for predicting death in advanced cancer patients within 3 days was 94.3% and 26.3%, respectively. In previous studies, the sensitivity of SQ to predict prognosis cancer patients within 12 months was 48.2–83.7%, and the specificity was 69.3–89.8%.^11^, ^12^, ^13^ Additionally, the sensitivity and specificity of the 30‐day Surprise Question proposed by Hamano et al., were 95.6% and 37%, respectively.^10^ Compared to these results, the 3DSQ had a higher sensitivity. The ability to avoid missing a patient who may die within 3 days is one of the 3DSQ's greatest strengths because families were reported to be most stressed during an unexpected death of a patient.^14^

One possible reason for the high sensitivity of the 3DSQ is the standardization of the questions to when the patient had PPS ≤20. A lot of physicians knew that patients with PPS ≤20 had a poorer prognosis. This may have caused the physicians to respond with "Not Surprised". PPS ≤20 is a physical sign that has a predictable prognosis within 3 days. However, its sensitivity was 64%,^9^ and the current study showed that 3DSQ was more sensitive. SQ is partly dependent on the physician's intuition ^15^ and this suggests that the sensitivity was increased by the addition of the physician's intuition based on the patient's condition (age, primary disease, symptoms, and general condition etc.) to the PPS≤20 physical signs. It is possible that the physician's intuition elicited by SQ may have increased sensitivity.

Based on the results of the SQ from the current and previous studies, the specificity tended to decrease from 69.3–89.8% at 12 months,^11^, ^12^, ^13^ 37% at 30 days,^10^ and 26.3% at 3 days. This implies that as the prediction period gets shorter, the SQ is less accurate in determining which patients will die.^10^ This may be the reason for the low specificity of the 3DSQ. However, it is important that the 3DSQ was found to be a highly sensitive prognostic tool in this study. If physicians answered "Not surprised" to the 3DSQ, the physicians had to ‘carefully explain to the patient's family the likelihood that the patient will die within 3 days. At the same time, the possibility of missing predictions due to the low specificity needed to be carefully explained as well. Consequently, we believe the 3DSQ is a useful tool for identifying patients who are likely to die within 3 days.

Physicians sometimes make wrong predictions that patients would live longer. This may be exhausting for the patient's families and damaging to the physician's relationship with them. When the patient is close to death, their family members hope to stay with the patients.^16^ However, in today's society, families are not always able to stay with their patients for as long as they would like because most of their families have to take time off from work and household chores and childcare to visit patients. In this study, we associated five variables with the “Lived longer group.” Pulselessness of radial artery, respiration with mandibular movement, and low oxygen saturation were known specific physical signs associated with death within 2 to 3 days.^17^ Thus, the absence of these signs predicts patient survival for more than 3 days. Patients who were given opioids were predicted to have poorer prognosis, and this may be related to delirium. Several studies proved that opioid administration was significantly associated with an increased risk of delirium.^18^ There were also reports that patients with hypoactive delirium were more common than expected in advanced cancer patients.^19^ Hence, the poor response of patients due to hypoactive delirium may have caused the poorer prognosis. This study revealed that patients who were not on continuous deep sedation were more likely to have long‐term survival, contrary to the physician's prognosis. The patients in the group that did not receive continuous deep sedation may have been in a poor general condition, so they did not need sedation.^20^, ^21^

This study has some limitations. First, the treatment plans differed from that of the general wards because the facilities were limited to cancer patients admitted to the palliative care units. However, it is unlikely that multidisciplinary therapy will be used for patients with advanced cancer in general wards especially in those with a PPS ≤20. Second, the physicians may have used other prognostic tools (i.e., PaP score, PPI, PiPS models, etc.) in clinical practice and their response to the 3DSQ may have been influenced by other prognostic tools. However, we consider that these prognostic tools do not affect this study because they were not designed to predict imminent death. Third, all assessments were performed by palliative care physicians. Therefore, studies involving physicians in other departments may yield different results in terms of sensitivity and specificity. In the future, we need to expand the scope of the study to include a wider range of diseases and institutions to confirm the usefulness of the 3DSQ.

## 5 CONCLUSION

The 3‐Day Surprise Question is a viable screening tool to identify advanced cancer patients with impending death, especially within 3 days.

## CONFLICT OF INTEREST

The authors declare that there is no conflict of interest.

## AUTHOR CONTRIBUTIONS

Tomoo Ikari: Conceptualization, Data curation, Formal analysis, Investigation, Methodology, Project administration, Writing—original draft, Writing—review and editing.

Yusuke Hiratsuka: Conceptualization, Data curation, Investigation, Methodology, —review and editing.

Takuhiro Yamaguchi: data curation, formal analysis, writing—review, and editing.

Isseki Maeda: Investigation, Writing—review and editing.

Masanori Mori: Conceptualization, Investigation, Methodology, Project administration, Resources, Supervision, Writing—review and editing.

Yu Uneno: Investigation, Writing—review and editing.

Tomohiko Taniyama: Investigation, Writing—review and editing.

Yosuke Matsuda: Investigation, Writing—review and editing.

Kiyofumi Oya: Investigation, Writing—review and editing.

Keita Tagami: Investigation, Writing—review and editing.

Akira Inoue: Conceptualization, Data curation, Investigation, Methodology, Project administration, Writing—original draft.

## FUNDING INFORMATION

EASED study was supported by a Grant‐in‐Aid from the Japanese Hospice Palliative Care Foundation.

## ETHICS STATEMENT

We conducted this study in accordance with the ethical standards of the Helsinki Declaration and the ethical guidelines for epidemiological research of the Ministry of Health, Labor and Welfare in Japan. All patients had access to the study information and were free to refuse to participate or secede. The local institutional review boards of all participating institutions approved the study.

The study was approved by the independent ethics committee of Tohoku University School of Medicine (approval no. 2016‐1‐689) and the local ethics committee of each participating center.

## ETHICAL APPROVAL AND CONSENT TO PARTICIPATE

Procedures performed in studies involving human participants were in accordance with the ethical standards of the independent ethics committee of Tohoku University School of Medicine (approval no. 2016‐1‐689) and with the 1964 Helsinki Declaration and its later amendments or comparable ethical standards. Written consent was unnecessary and patients were provided the opportunity to opt out.

## Supporting information

Table S1

## Data Availability

The data that support the findings of current study are available on request from the corresponding author.
