# Supplementary material for: “3‐Day Surprise Question” to predict prognosis of advanced cancer patients with impending death: Multicenter prospective observational study
Source: Cancer Med. 2020 Dec 21;10(3):1018–26. doi: 10.1002/cam4.3689 (PMC7897938; doi:10.1002/cam4.3689)
Supplement: Supplementary file 1 — Table S1 [file CAM4-10-1018-s001.docx]

**Table S1**. The result of all factors in univariate analysis.

| Total（n=1411） |  | Total | Lived longer group | | Other group | | p |
| --- | --- | --- | --- | --- | --- | --- | --- |
| Variables |  | n | n | % | n | % |  |
| Age | 20-59 year | 211 | 65 | 30.81 | 146 | 69.19 | 0.671 |
|  | 60-69 year | 328 | 141 | 42.99 | 187 | 57.01 |  |
|  | 70-79 year | 424 | 172 | 40.57 | 252 | 59.43 |  |
|  | 80-89 year | 369 | 136 | 36.86 | 233 | 63.14 |  |
|  | ≥90 year | 79 | 29 | 36.71 | 50 | 63.29 |  |
| Sex | Male | 716 | 289 | 40.36 | 427 | 59.64 | 0.155 |
|  | Female | 695 | 254 | 36.55 | 441 | 63.45 |  |
| Primary cancer site | Lung | 242 | 93 | 38.48 | 149 | 61.57 | 1.000 |
|  | Stomach/Esophagus | 207 | 90 | 43.48 | 117 | 56.52 | 0.122 |
|  | Colon/Rectum | 186 | 65 | 34.95 | 121 | 65.05 | 0.295 |
|  | Liver/Biliary system | 121 | 42 | 34.71 | 79 | 65.29 | 0.435 |
|  | Pancreas | 146 | 57 | 39.04 | 89 | 60.96 | 0.929 |
|  | Ovary/Uterus | 82 | 27 | 32.93 | 55 | 67.07 | 0.349 |
|  | Prostate/Bladder/Kidney/Testis | 102 | 42 | 41.18 | 60 | 58.82 | 0.598 |
| Liver metastasis | Absent | 846 | 342 | 40.43 | 504 | 59.57 | 0.074 |
|  | Present | 565 | 201 | 35.58 | 364 | 64.42 |  |
| Bone metastasis | Absent | 1031 | 387 | 37.54 | 644 | 62.46 | 0.241 |
|  | Present | 380 | 156 | 41.05 | 224 | 58.95 |  |
| Lung metastasis | Absent | 880 | 352 | 40 | 528 | 60 | 0.142 |
|  | Present | 530 | 191 | 36.04 | 339 | 63.96 |  |
| Cardiovascular comorbidity | Absent | 1329 | 515 | 38.75 | 814 | 61.25 | 0.483 |
|  | Present | 82 | 28 | 34.15 | 54 | 65.85 |  |
| Cerebrovascular comorbidity | Absent | 1299 | 502 | 38.65 | 797 | 61.35 | 0.687 |
|  | Present | 112 | 41 | 36.61 | 71 | 63.39 |  |
| Lung comorbidity | Absent | 1328 | 508 | 38.25 | 820 | 61.75 | 0.487 |
|  | Present | 83 | 35 | 42.17 | 48 | 57.83 |  |
| Diabetes comorbidity | Absent | 1227 | 467 | 38.06 | 760 | 61.94 | 0.417 |
|  | Present | 184 | 76 | 41.3 | 108 | 58.7 |  |
| Dementia comorbidity | Absent | 1286 | 498 | 38.72 | 788 | 61.28 | 0.565 |
|  | Present | 125 | 45 | 36 | 80 | 64 |  |
| History of mentalillness | Absent | 139 | 53 | 38.13 | 86 | 61.87 | 1.000 |
|  | Present | 1272 | 490 | 38.52 | 782 | 61.48 |  |
| Surgery | Not performed | 817 | 325 | 39.78 | 492 | 60.22 | 0.245 |
|  | Performed | 594 | 218 | 36.7 | 376 | 63.3 |  |
| Chemotherapy | Not receive | 544 | 204 | 37.5 | 340 | 62.5 | 0.746 |
|  | Receive (not within 1 month) | 740 | 292 | 39.46 | 448 | 60.54 |  |
|  | Receive (within 1 month) | 126 | 47 | 37.3 | 79 | 62.7 |  |
| Hormonal therapy | Not receive | 1397 | 538 | 38.51 | 859 | 61.49 | 1.000 |
|  | Receive | 14 | 5 | 35.71 | 9 | 64.29 |  |
| Radiation therapy | Not receive | 1400 | 539 | 38.5 | 861 | 61.5 | 1.000 |
|  | Receive | 11 | 4 | 36.36 | 7 | 63.64 |  |
| Oxygen therapy | Not receive | 959 | 378 | 39.42 | 581 | 60.58 | 0.291 |
|  | Receive | 451 | 164 | 36.36 | 287 | 63.64 |  |
| Presence of opioid administration | Not receive | 324 | 150 | 46.3 | 174 | 53.7 | 0.002 |
|  | Receive | 1072 | 393 | 36.7 | 679 | 63.3 |  |
| Eastern Cooperative Oncology Group Performance Status | From0 to 3 | 636 | 249 | 39.15 | 387 | 60.85 | 0.641 |
|  | 4 | 775 | 294 | 37.94 | 481 | 62.06 |  |
| Continuous deep sedation | No | 1349 | 534 | 39.6 | 815 | 60.4 | 0.006 |
|  | Yes | 47 | 9 | 19.2 | 38 | 80.8 |  |
| Richmond Agitation Sedation Scale score | From -5 to -2 | 667 | 246 | 36.9 | 421 | 63.1 | 0.124 |
|  | From -1 to 5 | 726 | 297 | 40.9 | 429 | 59.1 |  |
| Decreased response to verbal stimuli | No | 1172 | 477 | 40.7 | 695 | 59.3 | 0.002 |
|  | Yes | 224 | 66 | 29.5 | 158 | 70.5 |  |
| Decreased response to visual stimuli | No | 1012 | 420 | 41.5 | 592 | 58.5 | 0.001 |
|  | Yes | 384 | 123 | 32 | 261 | 68 |  |
| Peripheral cyanosis | No | 1146 | 466 | 40.7 | 680 | 59.3 | 0.004 |
|  | Yes | 250 | 77 | 30.8 | 173 | 69.2 |  |
| Pulselessness of radial artery | No | 1327 | 531 | 40 | 796 | 60 | <0.001 |
|  | Yes | 69 | 12 | 17.4 | 57 | 82.6 |  |
| Respiration with mandibular movement | No | 1346 | 540 | 40.1 | 806 | 59.89 | <0.001 |
|  | Yes | 50 | 3 | 6 | 47 | 94 |  |
| Increased bronchial secrretions | No | 1067 | 435 | 40.8 | 632 | 59.2 | 0.009 |
|  | Yes | 329 | 108 | 32.8 | 221 | 67.2 |  |
| Body temperature | Lower than 37.5 degrees | 947 | 374 | 39.49 | 573 | 60.51 | 0.422 |
|  | Above 37.5 degrees | 309 | 130 | 42.07 | 179 | 57.93 |  |
| SpO2 | 90% and above | 123 | 27 | 22 | 96 | 78 | <0.001 |
|  | 89% or less | 1162 | 492 | 42.2 | 674 | 57.8 |  |
| Respiratory rate | 24 times or less per minute | 1100 | 449 | 40.8 | 651 | 59.2 | 0.041 |
|  | 25 times or more per minute | 76 | 22 | 29 | 54 | 71 |  |
| Pain | IPOS (from 0 to 1) | 1110 | 430 | 38.7 | 680 | 61.3 | 0.811 |
|  | IPOS (from 2 to 4) | 286 | 113 | 39.5 | 173 | 60.5 |  |
| Dyspnea | IPOS (from 0 to 1) | 1098 | 439 | 39.9 | 659 | 60.1 | 0.111 |
|  | IPOS (from 2 to 4) | 298 | 104 | 34.9 | 194 | 65.1 |  |
| Fatigue | IPOS (from 0 to 1) | 505 | 201 | 39.8 | 304 | 60.2 | 0.901 |
|  | IPOS (from 2 to 4) | 555 | 223 | 40.18 | 332 | 59.82 |  |
| Edema | No | 566 | 225 | 39.8 | 341 | 60.2 | 0.615 |
|  | Yes | 830 | 318 | 38.3 | 512 | 61.7 |  |
| Symptoms related to pleural effusion | Absent | 1148 | 454 | 39.55 | 694 | 60.45 | 0.284 |
|  | Present | 248 | 89 | 35.9 | 159 | 64.11 |  |
| Symptoms related to ascites | Absent | 1180 | 461 | 39.07 | 719 | 60.93 | 0.759 |
|  | Present | 216 | 82 | 37.96 | 134 | 62.04 |  |
| Prediction of prognosis at PPS ≤ 20 | Within 7 days | 1077 | 404 | 37.5 | 673 | 62.5 | 0.166 |
|  | 8 days or more | 333 | 139 | 41.7 | 194 | 58.3 |  |
